# Ca^2+^ sparks and beyond

**DOI:** 10.52601/bpr.2024.240909

**Published:** 2024-10-31

**Authors:** Shi-Qiang Wang

**Affiliations:** 1 State Key Laboratory of Membrane Biology, College of Life Sciences, Peking University, Beijing 100871, China

The calcium ion (Ca^2+^) is the simplest but most versatile intracellular messenger, which regulates nearly all intracellular processes, from gene expression to protein modification, from fertilization to proliferation, from cell survival to cell death. Why Ca^2+^ fulfills so many functions? Why Ca^2+^ is involved in a variety of diseases? Answering these questions relies on the microscopic understanding of intracellular Ca^2+^ activities.

In 1993, Heping Cheng, when he was a Ph.D. student in W. Jonathan Lederer’s lab, for the first time identified an elementary intracellular Ca^2+^ event produced by one or several ryanodine receptor (RyR) Ca^2+^ release channels, which is named as a “Ca^2+^ spark”. The discovery of the Ca^2+^ spark has opened the new era to explore the microscopic aspect of Ca^2+^ signaling. Due to his achievements in Ca^2+^ and other intracellular signal studies, Prof. Heping Cheng was granted the eighth Shizhang Bei Award by the Chinese Society of Biophysics in 2023.

To highlight the 30-year anniversary of Ca^2+^ spark discovery and the contributions of Chinese scientists in the Ca^2+^ signaling field, the Editor-in-Chief Tao Xu suggested organizing this special issue and invited Prof. Cheng to write a topical review about Ca^2+^ signaling. Experts working on different aspects of Ca^2+^ signaling studies are also invited to contribute to this special issue to reflect the recent advances in this field.

As the first article of this issue, the review by Prof. Cheng and colleagues outlines the 30 years of achievements of Ca^2+^ signaling microdomains since the discovery of the “Ca^2+^ spark”, and postulates that a “digital” subsystem consisting of brief, high Ca^2+^ concentration over short distances (nanometers to microns) underlies the apparent “analog” global Ca^2+^ signaling (Lu *et al*. 2024). The spatiotemporal patterning of the digital Ca^2+^ signals underpins signaling efficiency, stability, specificity, and diversity of Ca^2+^-mediated biological processes.

The all-or-none open and close of Ca^2+^-permeable channels constitute the fundamental basis for the “digital” nature of local Ca^2+^ signals. These channels, no matter they are highly selective to Ca^2+^, such as the voltage-gated L-type Ca^2+^ channel (LCC), or less selective/nonselective channels, such as the transient receptor potential vanilloid (TRPV) channels and the calcium homeostasis modulator (CALHM) pores as reviewed by Prof. Yuquan Shen *et al*., contribute in distinct manners to the versatility of Ca^2+^ signaling (Ren *et al*. 2024). For example, the gap junction channels formed by head-to-head docking of two connexin hemichannels may conduct electrical and Ca^2+^ signals between neighboring cells. Prof. Donglin Bai and colleagues show here that, besides Ca^2+^, Mg^2+^ is also a key regulator of the rectification property of gap junction channels (Chen and Bai 2024). Compared with that in other tissues, Ca^2+^ channels in the heart are most intensively studied. During excitation of cardiomyocytes, openings of LCCs generate brief and weak initial Ca^2+^ signals “Ca^2+^ sparklets” in the microdomain between the cell/T-tubule membrane and the junctional sarcoplasmic reticulum (SR). The ryanodine receptors (RyRs) in the junctional SR respond to Ca^2+^ sparklets via the Ca^2+^-induced Ca^2+^ release mechanism, giving rise to numerous “Ca^2+^ sparks” that sum up to drive cell contraction. In sports or emergent situations, as reviewed by Prof. Huaqian Yang *et al*., both LCCs and RyRs respond to the sympathetic innervation with an increased opening probability such that both Ca^2+^ sparklets and Ca^2+^ sparks become stronger, which are then translated to stronger heart contraction to meet the blood pumping needs (Yang *et al*. 2024).

Ca^2+^ signals are supported by a variety of intracellular Ca^2+^ stores, including endoplasmic reticulum, SR, nucleus envelope, mitochondria, as well as the Golgi apparatus. Most of these Ca^2+^ stores contain proteins with strong Ca^2+^ buffering capacity, and the Ca^2+^ diffusion in the presence of heavy immobile Ca^2+^ buffers in these stores is expected to be slow. However, the simulation here by Prof. Wenjun Xie *et al*. demonstrated that Ca^2+^ diffuses very quickly inside local junctional SR cisternae (Su *et al*. 2024). They have also developed an assay to measure the SR Ca^2+^-leak, which is very helpful for studying the Ca^2+^ handling by the SR (Xu *et al*. 2024). Compared with the Ca^2+^ activity in SR, the activity and function of mitochondrial Ca^2+^ are less understood. Here Prof. Youjun Wang *et al.* developed a bright cyan fluorescence Ca^2+^ indicator to detect mitochondrial Ca^2+^ with improved performance (Gu *et al*. 2024). The role of Golgi apparatus as a Ca^2+^ store, as reviewed by Prof. Zongjie Cui, was firstly discovered by Prof. Shao Bai Xue in Beijing Normal University in the 1990s (Cui 2024). Later findings of Golgi Ca^2+^ handling proteins have outlined the whole picture of this unique Ca^2+^ store.

After coming to cytosol with different spatiotemporal patterning, Ca^2+^ fulfills distinct functions by binding to different intracellular targets and activating different signaling cascades. For example, Ca^2+^ is found as an important regulator of the extracellular regulated protein kinases (ERK), which is a core component of the mitogen-activated protein kinase (MAPK) signaling cascade implicated in cell proliferation and migration, as well as tumorigenesis and cell death. The ERK signaling also conversely regulates Ca^2+^ dynamics. Prof. Chailiang Wei *et al.* optimized the method to detect Ca^2+^ dynamic and ERK activity simultaneously, which is very useful for dissecting the causal characteristics of these two signals (Zhang *et al*. 2024).

Under physiological conditions, Ca^2+^ entry into the cytosol is balanced by the Ca^2+^ removal back to extracellular space and intracellular stores, a concept known as Ca^2+^ homeostasis. Dysregulation of Ca^2+^ signaling and homeostasis leads to a variety of cardiovascular, immune, neural and metabolic diseases. As reviewed by Ying Hu *et al.*, abnormal calcium handling underlies the out-of-control of proliferation, survival and invasion in cancer cells (Su *et al*. 2024). Therefore, targeting proteins in Ca^2+^ and Ca^2+^ homeostasis has become a main strategy in developing novel treatments against cancers and many other diseases.

## Conflict of interest

Shi-Qiang Wang declare that they have no conflict of interest.
